# A conceptual framework for selecting appropriate populations for public health interventions

**DOI:** 10.3389/fpubh.2023.1161034

**Published:** 2023-05-05

**Authors:** Jens Aagaard-Hansen, Anette Lykke Hindhede, Helle Terkildsen Maindal

**Affiliations:** ^1^Copenhagen University Hospital – Steno Diabetes Center Copenhagen (Health Promotion Research), Herlev, Denmark; ^2^SA MRC Developmental Pathways for Health Research Unit, Faculty of Health Sciences, University of the Witwatersrand, Johannesburg, South Africa; ^3^UCSF Centre for Health Research, Copenhagen University Hospital, Copenhagen, Denmark; ^4^Section for Health Services Research, Department of Public Health, University of Copenhagen, Copenhagen, Denmark; ^5^Section for Health Promotion, Department of Public Health, Aarhus University, Aarhus, Denmark

**Keywords:** interventions, life-course, population, public health, social determinants, space

## Abstract

This article suggests a conceptual framework for choice of target populations for public health interventions. In short, *who should benefit?* Taking the seminal work of Geoffrey Rose on “individuals at risk” versus the “whole population approach” as a point of departure, we explore later contributions. Frohlich and Potvin introduced the notion of “vulnerable populations” applying relevant social determinants as the defining selection criterion. Other interventions focus on a “physical space” (spatial demarcations) such as a neighborhood as a means to define intervention populations. As an addition to these criteria, we suggest that the life-course perspective entails an alternative means of selecting target populations based on a “temporal” perspective. A focus on the various age phases ranging from fetal life and infancy to old age may guide selection of population segments for targeted public health interventions. Each of the selection criteria has advantages and disadvantages when used for primary, secondary, or tertiary prevention. Thus, the conceptual framework may guide informed decisions in public health planning and research regarding precision prevention versus various approaches to complex community-based interventions.

## Introduction

Irrespectively of the public health issue in question, implicit or explicit choices are made regarding the target population for appropriate interventions. For instance, screening programs are based on the rationale of focusing on populations at risk defined by various biomedical markers; and health promotion interventions will emphasize various solutions with consequential age group, gender and potentially place implications. But there are other ways of defining *who* should benefit from a given intervention, i.e., which population segment should be targeted.

This paper explores the various criteria based on which target populations for public health interventions are chosen and suggests a conceptual framework based on four criteria—biomedical, social, spatial, and temporal. We contend that this may guide strategic choices within public health planning and research.

## Various criteria for population selection

### Whole populations or persons at risk?

**Figure 1:**
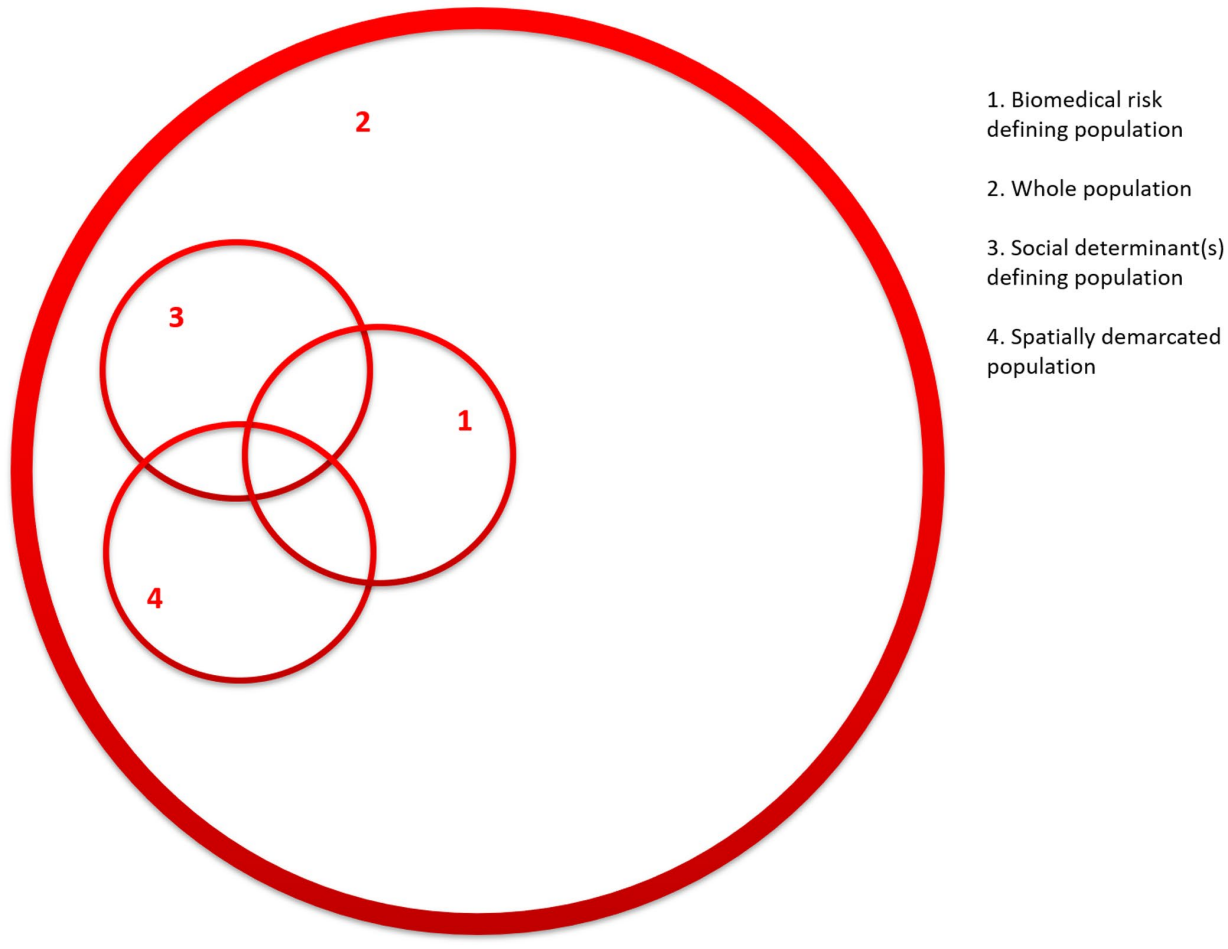
Population segments determined by various selection criteria. The numbers indicate: (1) biomedical criteria, (2) whole population approach, (3) social determinant(s) defining population, and (4) spatially demarcated population.

**Figure 2:**
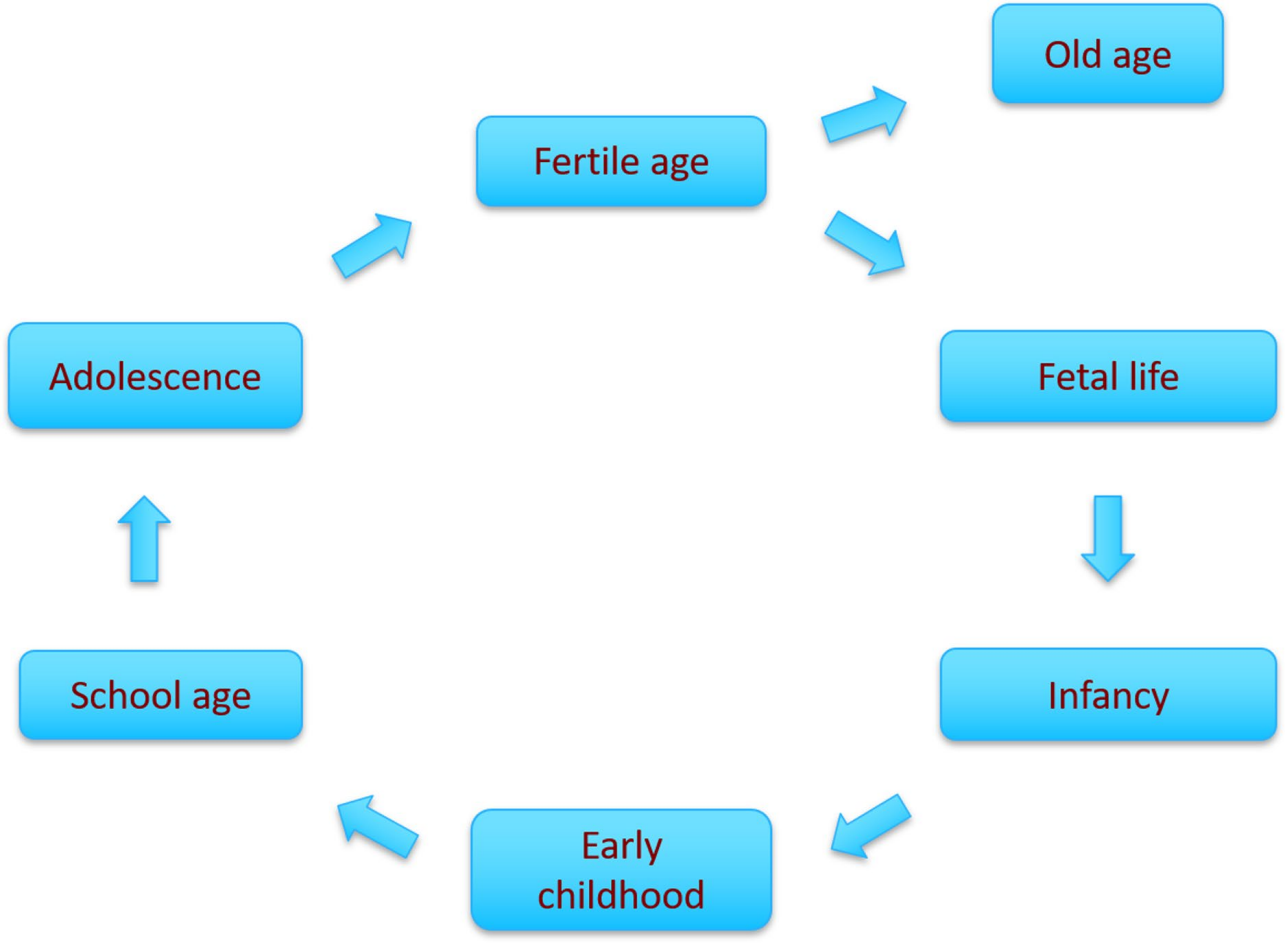
Circular representation of the main phases of the life-course, illustrating how each phase chronologically leads to the next with accumulated risk (20).

In 1974, the Canadian Lalonde report recommended a focus on “populations at risk” characterized by either risk behavior or biomarkers (1). Nine years later in 1985, Geoffrey Rose introduced the alternative “whole population approach” and interposed these two approaches to public health interventions (2). On the one hand many programs focus on a certain risk factor (e.g., smoking) or biomarker (e.g., elevated blood pressure identified through screening) thereby identifying a segment of the total population (Figure 1, 1) and subsequently implementing remedial actions. A classic example is the interventions screening for borderline elevated blood sugar (prediabetes) and subsequent comprehensive lifestyle programs (3). On the other hand, Rose pointed to the “whole population” perspective whereby a given intervention covers everyone, e.g., through fiscal policies or mandatory fortification of food with micronutrients such as iodizing of salt (Figure 1, 2) (4, 5). In essence, the former does relatively much for relatively few, whereas the latter leads to minor improvements for many. The point here is, that according to Rose the overall sum of the impact on the “whole population” is larger than the total impact on the group of “individuals at risk.” There are pros and cons of both approaches. A focus on individuals at risk concentrates the intervention resources where the immediate need is most conspicuous, but there is a danger of stigmatization, and it comes relatively late (secondary prevention). In contrast, a population approach targets everyone irrespectively of risk profile in what can be perceived more as primary prevention, but the fundamental root causes are not addressed, as mentioned below.

### Additional approaches

**Figure 3:**
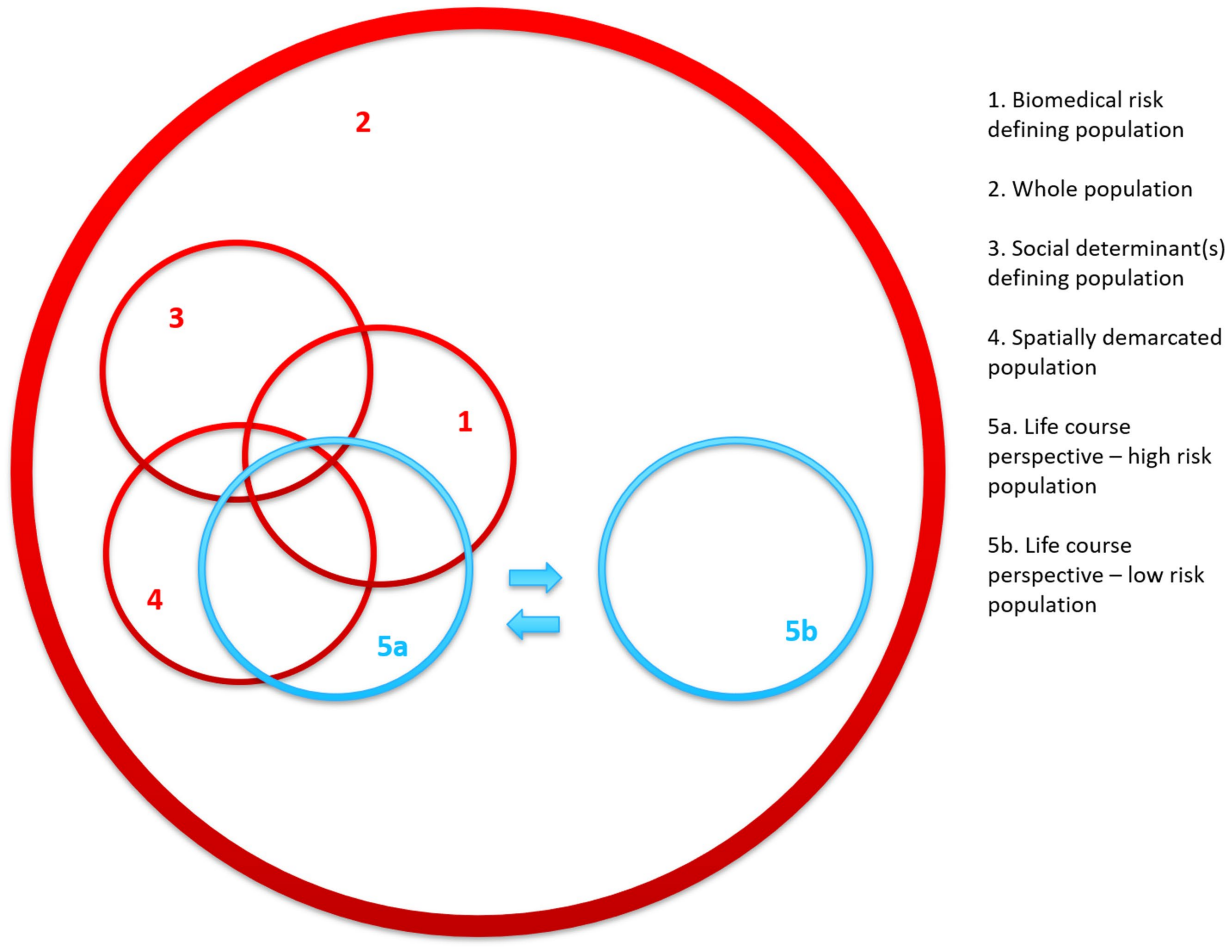
Conceptual framework illustrating population segments determined by various selection criteria with the addition of two examples of population selection based on a simplified version of the life-course perspective. The numbers indicate: (1) biomedical criteria, (2) whole population approach, (3) social determinant(s) defining population, (4) spatially demarcated population, (5a) age segment with high risk, and (5b) age segment with low risk.

Notwithstanding Rose’s insights, there have been critical voices. Based on the notion of “fundamental causes” (6), Frohlich and Potvin point out that a whole population approach does not address the underlying determinants, and that it is likely to increase health inequalities due to uneven distribution of risk factors as well as disparate ability to benefit from interventions (1). If for instance, poverty or low education level are social determinants for a given risk factor, they will remain unchanged even if this factor is addressed, and there may even be a situation where social disparities increase because the more well-to-do benefit more. As an alternative, they coined the concept of “vulnerable populations,” which they defined as a “subgroup or subpopulation who, because of shared social characteristics, is at higher risk of risks” (1, p. 218). Hence, a way to identify a target population is based on relevant social determinants (Figure 1, 3), but in terms of interventions the authors point out that the “vulnerable populations” should be complementary to the whole population approach (i.e., Figure 1, 2 and 3) (1).

Around the same time the extensive work of the Commission on Social Determinants of Health (7) and other sub-commissions (8) further strengthened this perspective by pointing out how health is unjustly and unevenly distributed in various population segments according to a number of social determinants, such as socio-economic status, education level, ethnicity or gender depending on local contexts just to mention a few. However, apart from this evidence bringing social injustice to the fore, it has a bearing for practice. As suggested by Frohlich and Potvin selected social determinants may be used to identify a population segment for which tailored public health interventions should be directed (Figure 1, 3) (1).

But there are other approaches to identifying populations at risk, for instance by applying a “spatial perspective” in which case a target population is demarcated by where they live, e.g., in a neighborhood or municipality (Figure 1, 4) (9, 10). There are several good reasons for this approach. Partly, neighborhoods are often characterized by particular risk profiles. Hence, some neighborhoods are inhabited by population segments with certain demographic and socio-economic profiles (as indicated by the overlap of 3 and 4 in Figure 1). Partly, a given neighborhood is likely to fall within the mandate of a given local administration, which may be engaged as a partner in public health interventions. Partly, a group of people living in the same local community often (but not always) has a sense of community and interact within social networks, which may work as a risk as well as an asset (11, 12). Finally, a given local community provides opportunities to engage in social interaction within the neighborhood with the aim to promote health based on social network analysis and identification of key opinion leaders (13–15).

### Adding the life-course perspective

For the past decades, biomedical research has highlighted the importance of the life-course perspective. Building on epidemiological and epigenetic research, the life-course concept describes how positive and negative exposures affect individuals’ cumulative risk profiles throughout the life-course with consequences for a wide range of diseases and socioeconomic and educational achievements (16–18). The life-course may be considered to span the period from conception to death. However, risk of disease accumulates not only throughout an individual’s life from the fetal stage onwards but is also passed on from one generation to the next (19). Consequently, one can visualize the notion of life-course as a circle incorporating each stage of life: fetal life, infancy, early childhood, school age, adolescence, and fertile age (including the preconception period). Within this circle, positive and negative events at any stage of the life-course may have an impact on subsequent stages and even on following generations. Old age is the exception, where seen from a biomedical viewpoint the impacts of events are not transmitted to the next generation (Figure 2) (20).

There is strong evidence that the exposures in early life, conceptualized as the “first 1,000 days” of the life-course (including fetal life and first years of infancy), are associated with later health trajectories, and researchers have even argued for an extended focus including adolescence (21, 22). Apart from this biomedical perspective on the life-course, there are important alternative psychological or sociological perspectives which are beyond the scope of the present article (23).

However, the life-course perspective is also relevant for public health interventions in the sense that it addresses root causes of disease and may guide selection of target population segments. In some cases, sub-populations defined as age brackets of the life-course will be characterized by a high-risk profile (Figure 3, 5a). If a program focuses on secondary prevention of non-communicable diseases, e.g., diabetes or cancer, it is likely that a focus on older adults will encompass a substantial part of individuals with relevant risk factors (Figure 3, overlap between 1 and 5a). Furthermore, there may be an over-representation among population segments characterized by certain social determinants (Figure 3, overlap between 3 and 5a), or within a certain physical location (Figure 3, overlap between 4 and 5a). Another example is Human Immunodeficiency Virus/Acquired Immune Deficiency Syndrome (HIV/AIDS) which is likely to peak in the age group of fertile (sexually active) age.

In contrast, there are other stages of the life-course, which demarcate different, low-risk population segment (Figure 3, 5b). For instance, a focus on early childhood development interventions is designated to a population segment with minimal acute health risks, but with a large prevention potential (24, 25). Another example is the recent focus on pre-conception interventions where timely action prior to pregnancy holds a larger prevention potential than interventions during pregnancy (26, 27). Even the temporal life-course-based approach may pose challenges. For instance, access to certain age groups may be impracticable. This is illustrated by the recent Malaysian Jom Mama trial aiming for reduced waist circumference among newlywed couples (28). The intervention combined an e-learning platform and six contacts with specially trained nurses. Despite careful tailoring and preparation, the project only managed to recruit 26% of the eligible persons due to high mobility and busy schedules, even though one would expect this group to be highly motivated on the brink to parenthood.

It should be noted that Frohlich and Potvin also highlighted the importance of the life-course perspective, though it was more to emphasize the accumulative character of risk during the life-course, rather than the notion of selection of target population (1).

## Discussion: from population selection to intervention

Looking at the various selection criteria of the conceptual framework presented in Figure 3, a certain pattern appears. Thus, populations defined by biomedical risk (Figure 1, 1, partially overlapping with 3, 4, and 5a) point to a potential for secondary or even tertiary prevention, whereas the whole population approach and the sub-population carved out by the early phases of the life-course (Figure 3, 2 and 5b) indicate domains of health promotion and primary prevention—especially 5b as it focuses on phases where the plasticity (physiological potential for change) is the largest. In between these two positions are the vulnerable population segments defined by social determinants (Figure 3, 3) or spatial criteria (Figure 3, 4), which may encompass sub-groups of all kinds, though the fraction of individuals at risk is likely to be higher than in the whole population in general.

It is a key element of public health interventions to address either individual, behavioral change (“lifestyle”) or structural, environmental living conditions or a combination thereof. Though it is beyond the scope of the present paper, it should be noted, that the various selection criteria mentioned above tend to entail different courses of action. Thus, a biomedical risk criterion (Figure 1, 1) almost automatically leads to interventions aimed at individual lifestyle factors (precision prevention). In contrast, a choice of a whole population (Figure 1, 2) or population selection based on either social determinant(s) or locality (Figure 1, 3 and 4) leads to structural interventions such as fiscal policies or complex modifications of physical environments or community-based interventions.

The suggested framework is not only of academic interest. We contend that a deliberate selection of intervention population segments will entail the best possible return of investment, which is essential within often resource-scarce health care systems in most parts of the world.

## Conclusion

It is an essential part of planning public health in general and health promotion interventions in particular, to identify the most appropriate target population. Usually this happens automatically because of the problem identified and habitual choices of intervention tools. However, we contend that there are choices to be made regarding target populations that all have pros and cons, which is why an in-depth analysis of the optimal population-level is required in the intervention planning phase. This article has presented a conceptual framework for selection of target population based on either biomedical, social, spatial, or temporal criteria, which may be of use to planning and research of public health interventions including health promotion and disease prevention.

## Data availability statement

The original contributions presented in the study are included in the article/supplementary material, further inquiries can be directed to the corresponding author.

## Author contributions

JA-H conceived the initial idea. All authors contributed to all phases of the writing and approved the final version.

## Conflict of interest

The authors declare that the research was conducted in the absence of any commercial or financial relationships that could be construed as a potential conflict of interest.

## Publisher’s note

All claims expressed in this article are solely those of the authors and do not necessarily represent those of their affiliated organizations, or those of the publisher, the editors and the reviewers. Any product that may be evaluated in this article, or claim that may be made by its manufacturer, is not guaranteed or endorsed by the publisher.
